# Plasma-derived exosomal miRNAs as potentially novel biomarkers for type 2 diabetes mellitus with abdominal obesity

**DOI:** 10.3389/fendo.2025.1656132

**Published:** 2025-11-28

**Authors:** Yan Li, Youqin Wang, Zhan Li, Lihui Feng, Ru Feng, Jia Jia, Li Xing, Lu Zheng

**Affiliations:** 1 Department of Endocrinology, Heping Hospital Affiliated to Changzhi Medical College, Changzhi, China; 2 The First Clinical College of Changzhi Medical College, Changzhi, China; 3 Changzhi Key Laboratory of Precision Medicine for Obesity and Metabolic Diseases, Changzhi, China

**Keywords:** abdominal obesity, type 2 diabetes, exosomal, microRNA, biomarkers

## Abstract

**Aims:**

To explore the plasma exosomal miRNA expression profiles in patients with abdominal obesity and type 2 diabetes mellitus (T2DM), identify differentially expressed exosomal m4RNAs, and evaluate the potential of selected exosomal miRNAs as biomarkers for abdominal obesity-related T2DM.

**Methods:**

In the screening phase, high-throughput sequencing was used to detect the plasma exosomal miRNA expression profiles of non-abdominal obese individuals with normal glucose metabolism (NAO/NG, N = 4), abdominal obese individuals with normal glucose metabolism (AO/NG, N = 4), and abdominal obese T2DM patients (AO/T2DM, N = 4). Five differentially expressed miRNAs were identified. In the independent validation phase, RT-qPCR was performed to validate the expression of these miRNAs in the NAO/NG group (N = 18), AO/NG group (N = 14), and AO/T2DM patients (N = 17). The correlation between the miRNA levels in AO/T2DM patients and clinical features was evaluated, and the diagnostic value of the selected miRNAs for AO/T2DM was analyzed using receiver operating characteristic (ROC) curves.

**Results:**

In the screening phase, 5 exosomal miRNAs showed sequential changes among the NAO/NG, AO/NG, and AO/T2DM groups. The AO/NG group specifically expressed 16 exosomal miRNAs, while the AO/T2DM group specifically expressed 7 exosomal miRNAs. In the independent validation phase, the expression of exosomal miRNAs hsa-let-7g-5p and PC-3p-13356 in plasma increased progressively across the NAO/NG, AO/NG, and AO/T2DM groups. The expression of exosomal hsa-miR-6505-5p in the AO/T2DM group was significantly higher than that in the AO/NG and NAO/NG groups. The expression of hsa-miR-1229-3p in plasma exosomes was significantly lower in the AO/NG group compared to the NAO/NG group, but higher in the AO/T2DM group compared to the AO/NG group. No differences were detected in the expression of hsa-miR-4750-5p among the groups. The areas under the ROC curves (AUC) for plasma exosomal hsa-let-7g-5p, PC-3p-13356, and hsa-miR-6505-5p were 0.857, 0.786, and 0.878, respectively. When combined, these three miRNAs showed high diagnostic value for abdominal obesity T2DM (AUC = 0.891).

**Conclusions:**

Patients with abdominal obesity and T2DM have a unique exosomal miRNA expression profiles. These characteristic exosomal miRNAs could serve as novel biomarkers for identifying such patients and may help in further understanding the pathogenesis of abdominal obesity-related T2DM.

## 1 Introduction

Diabetes mellitus is one of the fastest-growing global health challenges of the 21st century. It is estimated that currently, approximately 589 million adults aged 20–79 worldwide have diabetes mellitus, and this number is expected to surge to 850 million by 2050, with over 90% of these cases being Type 2 diabetes mellitus (T2DM) (1). The rapid rise in overweight/obesity rates is the main reason for the dramatic increase in global T2DM prevalence in recent years (2). Meta-analysis has found that the differences in visceral fat between populations with diabetes and without diabetes are much larger than those in subcutaneous fat (3). Compared to overall obesity, abdominal obesity is a stronger predictor of diabetes risk, and even in populations with a normal body mass index (BMI), abdominal obesity is still closely related to an increased risk of diabetes mellitus (4). Therefore, abdominal obesity plays a central role in the pathogenesis of T2DM. However, the molecular mechanisms by which abdominal obesity leads to T2DM are not fully understood, and current diagnostic markers for T2DM (such as OGTT, HbA1c, etc.) are limited by the disease itself and are insufficient to predict the onset or monitor its progression. Thus, there is an urgent need to further investigate the molecular mechanisms by which abdominal obesity affects T2DM and to explore new, clinically accessible, and efficient biomarkers.

Exosomes are extracellular vesicles secreted by cells, with a diameter of about 40–160 nm, and a widely distributed in various bodily fluids. They participate in cell communication and material transport by carrying bioactive molecules such as proteins, lipids, and nucleic acids (5). Among them, miRNAs, a class of abundant and functionally important RNA molecules in exosomes, have attracted significant attention. Thomou et al. (6) found that exosomal miRNA levels in the circulation of fat-specific Dicer (miRNA processing enzyme) knockout mice were significantly lower than in wild-type mice, while circulating miRNA levels showed no significant changes. This indicated that exosomal miRNA levels in circulation mainly depend on adipose tissue rather than the quantity of circulating miRNAs. This finding was further verified in patients with adipose tissue deficiency (7). It has also been found that in obesity, enlarged visceral adipocytes are more likely to secrete exosomes containing specific miRNAs. These exosomal miRNAs are novel signaling molecules that regulate the functions of distant organs such as the liver and pancreas, triggering inter-organ crosstalk and synergistic effects (8, 9). It plays a key regulatory role in the occurrence and development of metabolic disorders such as obesity and type 2 diabetes by inducing pancreatic β-cell damage, thereby exacerbating glucose intolerance and insulin resistance (IR) (10, 11). In addition, compared to other RNA molecules (such as lncRNA and mRNA), exosomal miRNAs not only have higher abundance and extraction efficiency but also exhibit better stability and specificity in tissues and blood (12). These characteristics make circulating exosomal miRNAs ideal biomarkers for T2DM. Dracheva et al. found (13) that serum exosomal miR-551b-3p expression was significantly higher in obese patients with T2DM compared to obese patients without T2DM. Receiver operating characteristic (ROC) analysis showed that exosomal miR-551b-3p had moderate predictive efficiency for obesity-related T2DM, suggesting that exosomal miR-551b-3p could serve as a potential biomarker of T2DM development in obesity. Furthermore, Kim et al. (14) studied the plasma exosomal miRNA profile in obese individuals, with or without T2DM. They found that exosomal miR-23a-5p, miR-6087, and miR-6751-3p showed dynamic, specific expression in healthy individuals, obese individuals without diabetes, and obese patients with diabetes. Specifically, the expression of miR-23a-5p and miR-6087 increased progressively, while miR-6751-3p expression decreased progressively. Since not all obese patients develop T2DM, the researchers believe these dynamically changing miRNAs may be involved in the pathological transition from obesity to T2DM and could be potential biomarkers for predicting obesity-related T2DM.

Recent research mainly focuses on systemic obesity, lacking studies specifically focusing on exosomal miRNAs in abdominal obesity-related T2DM. In this study, we not only describe the exosomal miRNA expression profiles in non-abdominal obese individuals with normal glucose metabolism, abdominal obese individuals with normal glucose metabolism, and abdominal obese individuals with newly diagnosed T2DM, but also preliminarily explores abdominal obesity-related T2DM-specific exosomal miRNAs. Additionally, our study evaluates the potential of these miRNAs as biomarkers to distinguish between abdominal obese individuals with normal glucose metabolism and abdominal obese individuals with T2DM, as well as their potential mechanisms, laying the foundation for further exploration of the pathogenesis of abdominal obesity-related T2DM.

## 2 Methods

### 2.1 Study design and participants

All subjects in this study were recruited from the Heping Hospital Affiliated to Changzhi Medical College. Based on the abdominal visceral fat area being ≥ 100 cm², subjects were divided into three groups: non-abdominal obesity with normal glucose metabolism (NAO/NG), abdominal obesity with normal glucose metabolism (AO/NG), and abdominal obesity with newly diagnosed T2DM (AO/T2DM) (**Table 1**). The T2DM diagnosis made using World Health Organization (WHO) guidelines published in 1999, with first-time diagnosis, no prior lifestyle changes, antihyperglycemic drugs, or insulin intervention, and no acute diabetes-related complications or severe liver and kidney dysfunction. The normal glucose metabolism group met the WHO 1999 criteria for normal glucose tolerance, had no family history of diabetes or history of hypertension, and had no abnormal liver or kidney function. This study was limited to individuals aged 18–60 years. Exclusion criteria for this study included:

**Table 1:** Clinical characteristics of participants in the screening phase.

Index	NAO/NG(n=4)	AO/NG(n=4)	AO/T2DM(n=4)	*p*
Age(years)	44.00 ± 3.55	39.75 ± 5.43	47.50 ± 3.31	0.080
Gender(Male/Female)	2/2	2/2	2/2	1.000
BMI(kg/m²)	25.07 ± 2.64	33.00 ± 6.43	27.7 ± 2.54	0.072
WC(cm)	84.90 ± 8.48	102.57 ± 19.26	91.20 ± 3.93	0.178
HC(cm)	96.37 ± 4.48	108.70 ± 12.51	95.75 ± 5.18	0.092
WHtR	0.53 ± 0.05	0.62 ± 0.11	0.57 ± 0.03	0.268
VFA(cm²)	76.75 ± 15.73	179.50 ± 55.97^a^	136.25 ± 11.06^a^	0.007
Systolic blood pressure(mmHg)	115.00 ± 14.67	130.75 ± 7.93	128.50 ± 22.94	0.379
Diastolic blood pressure(mmHg)	67.00 ± 9.31	82.25 ± 2.75	76.00 ± 18.24	0.245
FPG(mmol/L)	5.25 ± 0.37	5.56 ± 0.32	13.61 ± 2.34^ab^	0.003
HbA1c(%)	4.67 ± 0.25	4.77 ± 0.22	11.90 ± 1.46^ab^	0.001
TG(mmol/L)	1.65 ± 0.72	2.17 ± 0.61	2.52 ± 0.75	0.261
TC(mmol/L)	4.61 ± 1.25	4.61 ± 0.76	5.34 ± 0.54	0.456
LDL-C(mmol/L)	2.84 ± 0.84	3.07 ± 0.12	3.49 ± 0.32	0.263
HDL-C(mmol/L)	1.11 ± 0.24	1.00 ± 0.32	1.01 ± 0.14	0.778
ALT(U/L)	19.00 ± 3.56	37.00 ± 23.15	28.25 ± 15.97	0.342
AST(U/L)	22.50 ± 2.65	40.50 ± 28.60	24.75 ± 14.45	0.369
BUN(mmol/L)	4.15 ± 0.80	4.59 ± 1.63	4.99 ± 1.36	0.679
CREA(umol/L)	69.50 ± 18.84	69.00 ± 17.34	58.50 ± 12.92	0.588

The data are presented as the mean ± SD. NAO/NG, Non-abdominal obesity with normal glucose metabolism group; AO/NG, Abdominal obesity with normal glucose metabolism group; AO/T2DM, Abdominal obesity with type 2 diabetes mellitus group; BMI, Body Mass Index; WC: Waist Circumference; HC, Hip Circumference; WHtR, Waist-to-Height Ratio; VFA, Visceral Fat Area; FPG, Fasting plasma glucose; HbA1c, Glycated hemoglobin; TG, Triglycerides; TC: Total cholesterol; HDL-C: High-density lipoprotein cholesterol; LDL-C, Low-density lipoprotein cholesterol; ALT, Alanine aminotransferase; AST, Aspartate aminotransferase; BUN, Blood urea nitrogen; CREA, Creatinine; ^a^*p <* 0.05 (*vs* NAO/NG); ^b^*p <* 0.05 (*vs* AO/NG).

1. Prediabetes, type 1 diabetes mellitus, gestational diabetes, or other specific types of diabetes.
2. Coexisting autoimmune diseases, severe infectious diseases, severe organic diseases (e.g., myocardial infarction, stroke), or wasting diseases affecting body weight (e.g., malignant tumors, tuberculosis).
3. Pregnant or breastfeeding women, individuals with a history of weight loss in the past six months, or those taking medications that affect weight and energy expenditure.
4. Recent use of steroids, antipsychotic medications, or other drugs that may cause rapid increases in blood glucose levels.
5. Obesity caused by other factors such as medications, polycystic ovary syndrome, Cushing’s syndrome, pituitary tumors, or hypothyroidism.

This study was approved by The Ethics Committee of Heping Hospital Affiliated to Changzhi Medical College (Ethics approval number: (2024) 073), and all experiments adhered to the ethical principles of the Helsinki Declaration. All research participants signed informed consent forms.

This study consisted of two phases: a screening phase and a validation phase. In the screening phase, 4 gender- and age-matched subjects from each group (NAO/NG, AO/NG, and AO/T2DM) were selected for miRNA expression profile analysis using high-throughput sequencing (**Table 1**). In the validation phase, 18 age- and gender-matched subjects from the NAO/NG group, 14 from the AO/NG group, and 17 from the AO/T2DM group (**Table 2**) were included for candidate miRNA sequence verification using reverse transcription quantitative real-time PCR (RT-qPCR).

**Table 2:** Clinical characteristics of participants in the validation phase.

Index	NAO/NG (n=18)	AO/NG (n=14)	AO/T2DM (n=17)	*p*
Age(year)	42.44 ± 7.84	41.64 ± 6.11	46.18 ± 8.66	0.213
Gender (Male/Female)	13/5	8/6	9/8	0.471
BMI(kg/m²)	23.95(22.08,26.03)	30.80(27.88,32.40)^a^	28.50(26.25,29.50)^a^	<0.001
WC(cm)	81.76 ± 7.40	97.03 ± 9.75^a^	93.81 ± 6.72^a^	<0.001
HC(cm)	93.10(90.55,99.38)	103.00(99.58,108.98)^a^	100.00(95.65,104.45)^a^	<0.001
WHtR	0.51 ± 0.04	0.59 ± 0.05^a^	0.56 ± 0.03^a^	<0.001
VFA(cm²)	59.94 ± 12.89	142.07 ± 31.76^a^	134.29 ± 19.65^a^	<0.001
Systolic blood pressure(mmHg)	115.33 ± 12.52	122.43 ± 10.52	126.71 ± 12.33a	0.024
Diastolic blood pressure(mmHg)	70.50(57.00,75.25)	77.50(73.25,80.00)	93.10(90.55,99.38)	0.081
FBG(mmol/L)	5.24 ± 0.34	5.02 ± 0.49	7.97 ± 1.16^ab^	<0.001
HbA1c(%)	5.57 ± 0.34	5.46 ± 0.39	8.41 ± 1.37^ab^	<0.001
FINS(μIU/ml)	8.14 ± 3.80	12.94 ± 5.24^a^	12.09 ± 4.52^a^	0.008
HOMA-IR	1.91 ± 0.92	2.92 ± 1.33^a^	4.23 ± 1.50^ab^	<0.001
HOMA-β	93.48 ± 41.83	185.89 ± 91.77^a^	57.98 ± 26.13^ab^	<0.001
TG(mmol/L)	1.12(0.86,1.44)	1.49(1.30,2.16)	1.61(1.33,2.61)^a^	0.005
TC(mmol/L)	4.31(3.70,4.78)	4.13(3.43,4.96)	5.09(4.30,5.73)^ab^	0.006
LDL-C (mmol/L)	2.21 ± 0.44	2.50 ± 0.55	3.16 ± 0.82^ab^	<0.001
HDL-C (mmol/L)	1.32 ± 0.24	1.12 ± 0.18^a^	1.16 ± 0.22^a^	0.022
ALT(U/L)	17.34(11.30,24.59)	25.00(15.75,29.57)	35.00(28.00,61.00)^ab^	<0.001
AST(U/L)	21.00(18.02,23.25)	22.50(19.69,28.00)	32.00(23.00,36.00)^a^	0.002
BUN(mmol/L)	5.08 ± 1.22	4.29 ± 1.54	5.17 ± 1.01	0.119
CREA(umol/L)	61.46 ± 11.73	60.74 ± 10.69	60.56 ± 11.58	0.971

The data are presented as the mean ± SD, median (25th–75th percentile). NAO/NG: Non-abdominal obesity with normal glucose metabolism group; AO/NG: Abdominal obesity with normal glucose metabolism group; AO/T2DM: Abdominal obesity with type 2 diabetes mellitus group; BMI: Body Mass Index; WC: Waist Circumference; HC: Hip Circumference; WHtR: Waist-to-Height Ratio; VFA: Visceral Fat Area; FBG: Fasting blood glucose; HbA1c: Glycated hemoglobin; FINS: Fasting insulin; HOMA-IR: Homeostasis model assessment of insulin resistance; HOMA-β: Homeostasis model assessment of insulin secretion; TG: Triglycerides; TC: Total cholesterol; HDL-C: High-density lipoprotein cholesterol; LDL-C: Low-density lipoprotein cholesterol; ALT: Alanine aminotransferase; AST: Aspartate aminotransferase; BUN: Blood urea nitrogen; CREA: Creatinine; ^a^*p* < 0.05 (*vs* NAO/NG); ^b^*p* < 0.05 (*vs* AO/NG).

### 2.2 Anthropometric parameters and biochemical indicators detection

Clinical data, including name, gender, age, family history, medication history, and past medical history, were collected by fixed clinical physicians. Anthropometric parameters, including height, weight, and waist circumference, were measured following the Guideline for diagnosis and treatment of obesity (2024 edition) (15). The BMI was calculated as weight (kg) divided by height (m) squared, and the Waist-to-Height Ratio (WHtR) was calculated as the ratio of waist circumference to height. Blood pressure was recorded in mmHg while the participant was at rest, and the average of three measurements was used. Visceral Fat Area (VFA) was measured using a visceral fat analyzer (Omron HDS-2000, Japan) in a fasting, resting state. Biochemical indicators, including blood glucose, glycated hemoglobin (HbA1c), fasting insulin (FINS), triglycerides (TG), total cholesterol (TC), high-density lipoprotein cholesterol (HDL-C), low-density lipoprotein cholesterol (LDL-C), aspartate aminotransferase (AST), alanine aminotransferase (ALT), creatinine (CREA), and blood urea nitrogen (BUN), were detected in the laboratory of the Affiliated Peace Hospital of Changzhi Medical College. The Homeostasis Model Assessment of Insulin Resistance (HOMA-IR) was calculated as: HOMR−IR=FINSμIU/ml×FBGmmol/L/22.5; and the Homeostasis Model Assessment of Insulin Secretion (HOMA-β) was calculated as: HOMA−β=20×FINSμIU/ml/FBGmmol/L−3.5

### 2.3 Exosome extraction and identification

Fasting plasma samples were collected from patients, and exosomes were extracted using ultracentrifugation, following the method of Ye et al. (16) Western blot, Transmission electron microscopy (TEM), and nanoparticle tracking analysis (NTA) were used to verify the exosomes. The total protein of exosomes were extracted by using RIPA buffer. Protein quantification was performed using a BCA assay kit (Beyotime, China) according to the manufacturer’s instructions. Western blot analysis was performed to determine the expression of CD9 (Wuhan Sanying, China, 1:2000)、CD81 (Wuhan Sanying, China, 1:1000)、Tsg101 (Wuhan Sanying, China, 1:2000)、Calnexin (Wuhan Sanying, China, 1:5000)、FABP4 (Santa Cruz, America, 1:500)、β-actin (Wuhan Sanying, China, 1:4000). For transmission electron microscopy (TEM) analysis, the exosomes were fixed with 2% paraformaldehyde and loaded onto Formvar- and carbon-coated copper grids. The grids were then placed on 50 μl of 1% glutaraldehyde droplets for 5 min. After rinsing with ddH2O, the grids were placed on 50 μl of uranium dioxyoxalate droplets. After 5 min, the grids were placed on 50 μl of methyl cellulose droplets for 10 min. After drying for 5 min, the grids were visualized using the HT7700 transmission electron microscope (Hitachi, Japan). Furthermore, the particle size distribution of exosomes was measured by NTA using ZetaView_Particle Metrix (Particle Metrix,PMX-120, Germany) according to the instruments.

### 2.4 Total RNA extraction and RNA sequencing

Total RNA was extracted from plasma exosomes using the Exosomal RNA Isolation Kit (Norgen Biotek, Canada). The extraction procedure was strictly followed according to the kit instructions. RNA concentration was determined using a NanoDrop 2000 spectrophotometer (Thermo Scientific, USA). The extracted RNA was used as input material to construct the sequencing library using the Small RNA Sample Prep Kit. Index codes were added, and after passing the quality check of the library, sequencing was performed using the Illumina HiSeq2500 SE500 single-end sequencing.

### 2.5 miRNA analysis

Raw read data generated by Illumina HiSeq2500 SE500 single-end sequencing were processed using the ACGT101-miR software. High-quality sequences (clean reads), with the 3’ adapter removed, were obtained, and length filtering was performed to retain sequences ranging from 18 to 26 nucleotides. These remaining sequences were then aligned with sequences from various RNA databases, such as the Rfam database (containing rRNA, tRNA, snRNA, snoRNA, etc.) and the Repbase database (repetitive sequence database), resulting in valid data. To exclude non-miRNA sequences, a comparison was made with the Rfam database (version 12.0) and the Repbase database (version 22.07, repetitive sequence database). Sequences matching human miRNAs were preferentially retained based on miRNA conservation, prioritizing species, with human hsa sequences being prioritized first, against the miRBase database (version 22.0). Subsequently, secondary validation was performed using the GENCODE database to ensure that all included sequences matched human genome annotations. All miRNA data from non-human species (e.g., mmu, oga, bta) were excluded to prevent cross-species sequence contamination. The specific experimental process was assisted by Hangzhou Lianchuan Biotechnology Co., Ltd.

### 2.6 Real-time quantitative PCR analysis

Five candidate miRNAs were selected based on |log_2_fold change (FC)| > 1, a *p* – value < 0.05, and relevant literature: hsa-let-7g-5p, PC-3p-13356, hsa-miR-6505-5p, hsa-miR-1229-3p, and hsa-miR-4750-5p. RNA was reverse transcribed to cDNA using a miRNA first-strand DNA synthesis kit (Sangon Biotech (Shanghai) Co., Ltd., China). Subsequently, the RT-qPCR fluorescence quantification kit (Novogene, Nanjing, China) was used to measure the expression levels of the target miRNAs. The reaction conditions were as follows: 95°C for 5 min; [95°C for 10 s, 60°C for 30 s] repeated for 40 cycles; 95°C for 15 s, 60°C for 1 min; 95°C for 15 s, 60°C for 15 s. The primers for miRNA and the internal reference U6 were synthesized by GeneWiz (**Supplementary Table 1**). Relative quantification of candidate miRNA expression was performed using the 2^−ΔΔCt^ method. Each sample was tested in triplicate.

### 2.7 GO and KEGG enrichment analysis

Subsequently, we selected miRNAs consistent with the screening and validation results to predict their target genes and associated pathways. Target gene prediction for exosomal miRNAs was performed using the TargetScan (v5.5) and miRanda (v3.3a) databases. The analysis was conducted through the OmicStudio tool (https://www.omicstudio.cn/analysis/targetGene?id=4). Subsequently, Gene Ontology (GO) and Kyoto Encyclopedia of Genes and Genomes (KEGG) enrichment analyses were performed to explore the potential biological functions and pathways associated with the target genes.

### 2.8 PPI network construction and hub gene analysis

To better investigate the interactions among the target genes of differentially expressed miRNAs, we selected validated three miRNAs (hsa-let-7g-5p, PC-3p-13356, and hsa-miR-6505-5p) to construct a Protein-Protein Interaction (PPI) network. STRING database was used for the analysis, and Cytoscape software was employed for visualization. Hub gene analysis was performed using the Cytohubba plugin in Cytoscape software to identify key genes involved in the network.

### 2.9 Statistical analysis

Statistical analysis was conducted using SPSS 26.0 software. Normally distributed continuous data were expressed as mean ± standard deviation (Mean ± SD), and one-way analysis of variance (ANOVA) was used for comparisons among the three groups. *Post-hoc* pairwise comparisons were performed using LSD (Least Significant Difference) test. Non-normally distributed data were presented as median and interquartile range (P25~P75), and non-parametric Kruskal-Wallis test was used for pairwise group comparisons. Categorical data were expressed as frequencies, and chi-square (χ²) tests were applied for comparisons. Spearman correlation analysis was used to assess the relationships between variables. ROC curve analysis was performed to validate the diagnostic value of differentially expressed miRNAs. A *p* - value of < 0.05 was considered statistically significant.

## 3 Results

### 3.1 Identification of plasma exosomes

**Figure 1:**
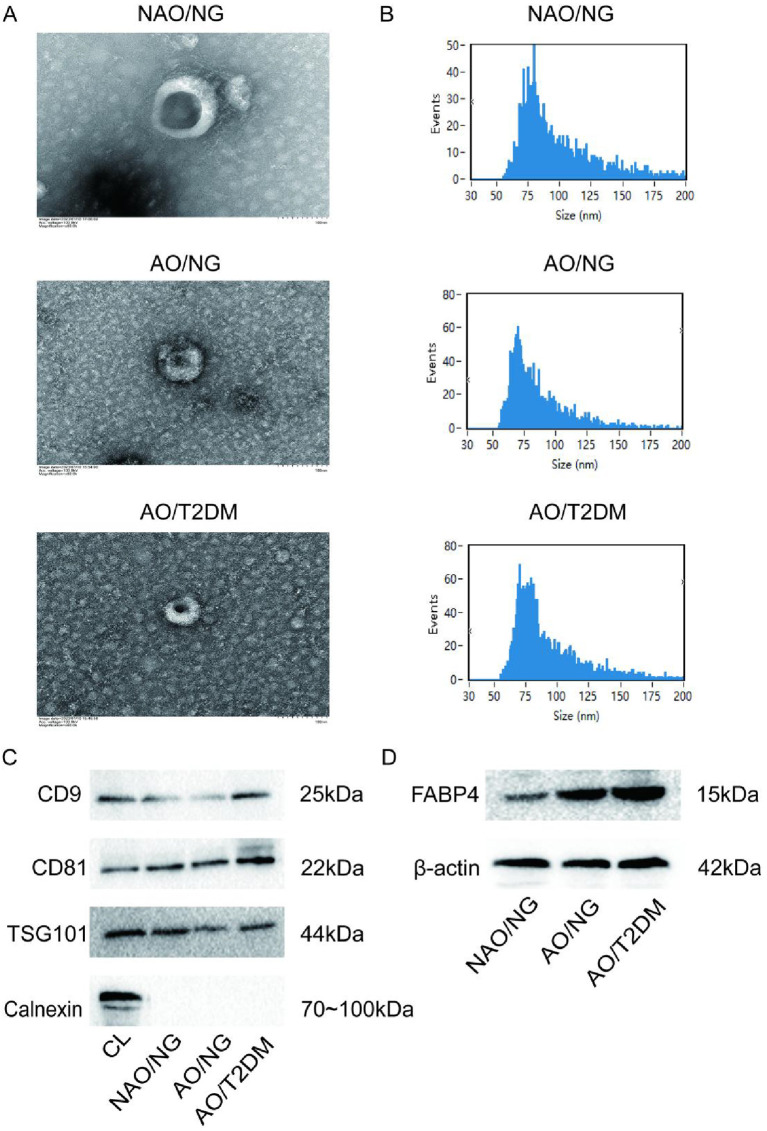
Exosome identification. The exosomes were analyzed by **(A)** TEM and NTA assay **(B)**. **(C, D)** The protein expression of CD9, CD81, TSG101, Calnexin, and FABP4 in exosomes was detected by Western blot assay. Cell lysate (CL) was used as a positive control for calmodulin.

TEM showed that exosomes from all three groups exhibited a “tea-tray” shaped, bilayer lipid membrane structure with intact morphology (**Figure 1A**). NTA revealed that the particle size distribution of exosomes from all three groups ranged from 50 to 200 nm, with an average size of approximately 90 nm, consistent with the characteristics of exosomes (**Figure 1B**). Western blot analysis comparatively examined the protein composition in whole cell lysates (CL) and plasma-derived exosomes. The samples of exosomes were found to be positive for the exosome markers, CD9, CD81, and TSG101, and negative for the endoplasmic reticulum marker Calnexin (**Figure 1C**). These results confirm the presence and proper preparation of plasma-derived exosomes.

To determine the potential origin of the exosomes, we further assessed the expression of the adipose tissue-specific protein-Fatty Acid Binding Protein 4 (FABP4) in the exosomes. The results showed that exosomes from all three groups contained FABP4 (**Figure 1D**), suggesting that the exosomes extracted in this study may originate from adipose tissue.

### 3.2 Plasma-derived exosomal miRNA analysis

In the screening phase, a total of 12 plasma-derived exosome samples from the NAO/NG, AO/NG, and AO/T2DM groups were analyzed. Using |log_2_(FC)| > 1 and a *p -* value < 0.05 as selection criteria, we found significant differential expression of 23 exosomal miRNAs in the AO/NG group compared to the NAO/NG group. Of these, 20 miRNAs were upregulated, and 3 were downregulated. In the AO/T2DM group, 8 exosomal miRNAs were significantly differentially expressed compared to the NAO/NG group, with 4 upregulated and 4 downregulated. Compared to the AO/NG group, 14 exosomal miRNAs were significantly differentially expressed in the AO/T2DM group, with 2 upregulated and 12 downregulated.

Based on this, we followed the method described by Kim et al. (14) (**Supplementary Figure 1**) to further screen for exosomal miRNAs specifically expressed in the AO/NG group. The results revealed 16 exosomal miRNAs that were specifically expressed in the AO/NG group, with 13 upregulated and 3 downregulated (**Supplementary Table 2**). Similarly, we used the same approach to screen for exosomal miRNAs specifically expressed in the AO/T2DM group (**Supplementary Figure 2**). We found 7 exosomal miRNAs that were specifically expressed in the AO/T2DM group, with 3 upregulated and 4 downregulated (**Supplementary Table 3**).

Finally, we analyzed the exosomal miRNAs that were sequentially upregulated or downregulated across the NAO/NG, AO/NG, and AO/T2DM groups. The results showed that there were 5 exosomal miRNAs with differential expression across the three groups, with 4 miRNAs being sequentially upregulated and 1 miRNAs being sequentially downregulated (**Supplementary Table 4**).

### 3.3 Validation and functional analysis of differentially expressed exosomal miRNAs

**Figure 2:**
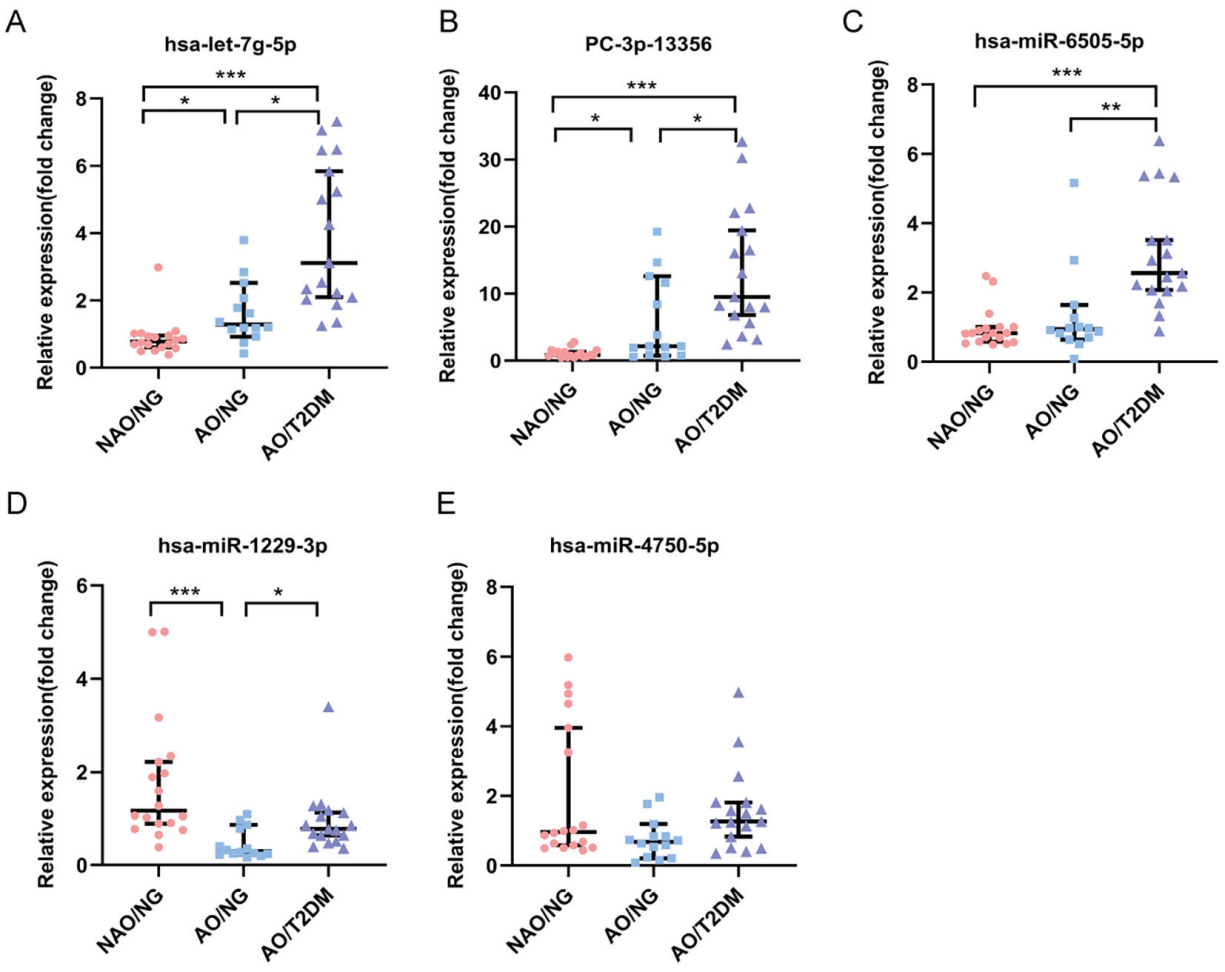
Comparison of the expression levels of miRNAs in plasma exosomes among three Groups **(A-E)**. *p < 0.05, **p < 0.01, ***p < 0.001.

Based on the criteria of |log_2_ (FC)| > 1, a *p* - value < 0.05, and related literature, we selected five miRNAs for sequence validation: hsa-let-7g-5p, PC-3p-13356, hsa-miR-6505-5p, hsa-miR-1229-3p, and hsa-miR-4750-5p. The results showed that the expression of plasma exosomal hsa-let-7g-5p and PC-3p-13356 sequentially increased from the NAO/NG group to the AO/NG group and then to the AO/T2DM group, with statistically significant differences between the groups (*p* < 0.05). The expression of plasma exosomal hsa-miR-6505-5p was significantly higher in the AO/T2DM group than in the AO/NG and NAO/NG groups (*p* < 0.01), while no statistically significant difference was observed between the AO/NG and NAO/NG groups (*p* > 0.05). The expression of plasma exosomal hsa-miR-1229-3p was significantly downregulated in the AO/NG group compared to the NAO/NG group (*p* < 0.01), but was significantly upregulated in the AO/T2DM group compared to the AO/NG group (*p* < 0.01). However, no statistically significant difference was observed between the AO/T2DM and NAO/NG groups (*p* > 0.05). In Addition, there were no differences in the expression of plasma exosomal hsa-miR-4750-5p among the three groups (*p* > 0.05). The expression changes of hsa-let-7g-5p, PC-3p-13356, and hsa-miR-6505-5p were consistent with the previous sequencing results (**Figure 2**, **Supplementary Table 5**).

### 3.4 Correlation between plasma-derived exosomal miRNAs and clinical parameters

The correlation analysis between plasma exosomal miRNAs and clinical parameters revealed the following results (**Supplementary Figure 4**): Exosomal PC-3p-13356 showed a positive correlation with WHtR, VFA, FBG, HbA1c, HOMA-IR, TG, and LDL-C, and a negative correlation with HDL-C. Exosomal hsa-let-7g-5p exhibited a positive correlation with WHtR, VFA, FBG, HbA1c, HOMA-IR, and LDL-C, and a negative correlation with HDL-C and HOMA-β. Exosomal hsa-miR-6505-5p was positively correlated with WHtR, VFA, FBG, HbA1c, HOMA-IR, and LDL-C, and negatively correlated with HOMA-β.

### 3.5 Exploration of plasma-derived exosomal miRNAs as biomarkers for abdominal obesity-related type 2 diabetes

**Figure 3:**
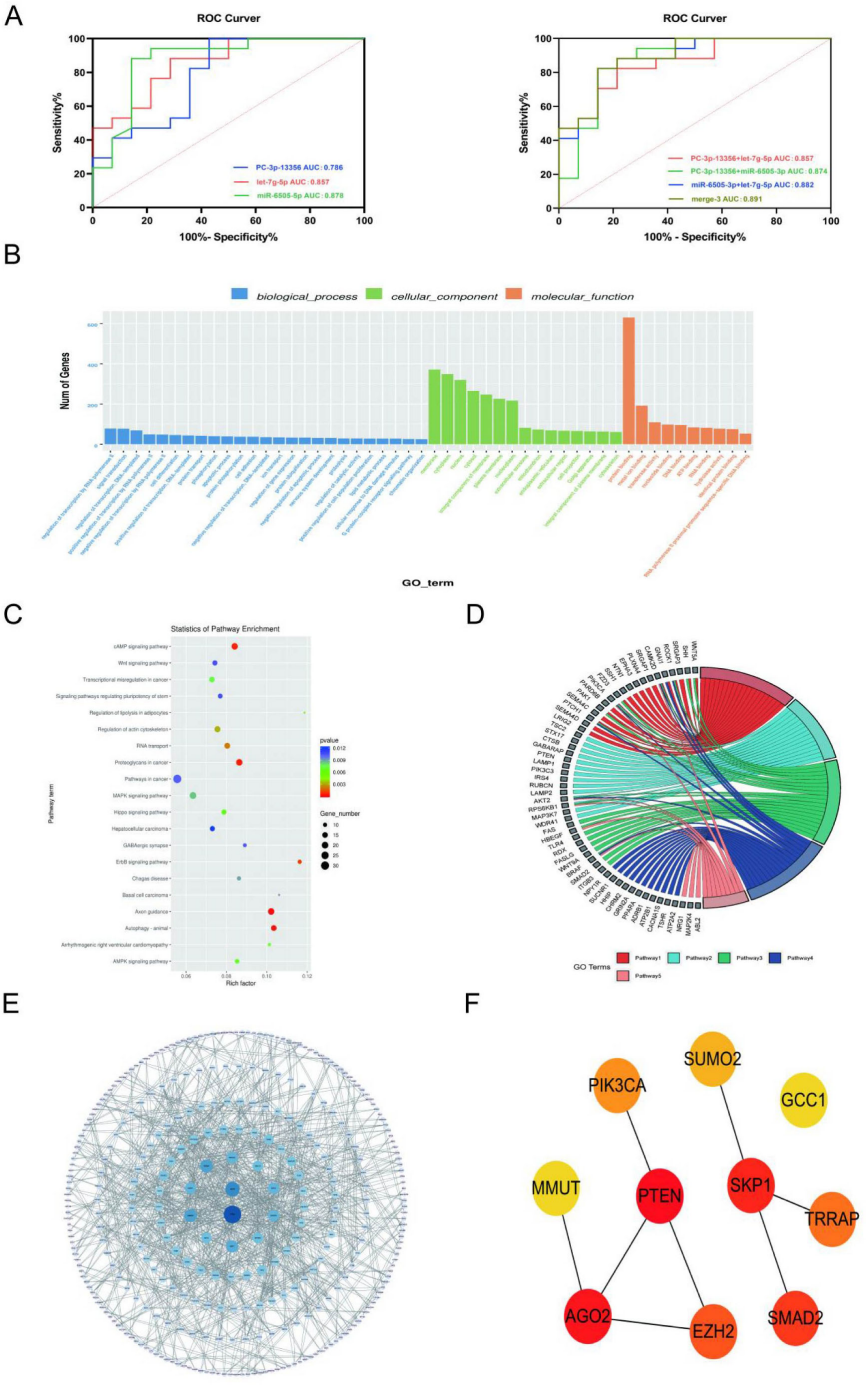
Plasma exosomal miRNAs as biomarkers for abdominal obesity-associated type 2 diabetes mellitus. **(A)** ROC curve analysis of plasma exosomal miRNAs. **(B)** Bar chart of GO analysis for target genes. **(C)** Bubble Chart of KEGG Pathway Analysis for Target Genes. **(D)** Chord diagram of the correlation between target genes and KEGG pathways. **(E)** PPI network diagram of the target genes of exosomal hsa-let-7g-5p, PC-3p-13356 and hsa-miR-6505-5p. **(F)** The main hub genes in the PPI network of the target genes of exosomal hsa-let-7g-5p, PC-3p-13356 and hsa-miR-6505-5p.

We further evaluated the potential of plasma exosomal miRNAs, namely hsa-let-7g-5p, PC-3p-13356, and hsa-miR-6505-5p, to distinguish between the AO/NG (abdominal obesity with normal glucose) group and the AO/T2DM (abdominal obesity with newly diagnosed T2DM) patients. The AUC (Area Under the Curve) values for exosomal miRNAs were as follows: hsa-let-7g-5p: 0.857, PC-3p-13356: 0.786, hsa-miR-6505-5p: 0.878. When combined in diagnostic models, the AUC values were improved: PC-3p-13356 + hsa-let-7g-5p: AUC = 0.857; PC-3p-13356 + hsa-miR-6505-5p: AUC = 0.874; hsa-miR-6505-5p + hsa-let-7g-5p: AUC = 0.882. The highest AUC of 0.891 was achieved in the model combining all three miRNAs (**Figure 3A**). Therefore, we propose that the quantification of plasma exosomal hsa-let-7g-5p, PC-3p-13356, and hsa-miR-6505-5p can be used as a primary or at least an auxiliary criterion for the diagnosis of Abdominal Obesity-Related Type 2 Diabetes.

Then we performed GO (Gene Ontology) and KEGG (Kyoto Encyclopedia of Genes and Genomes) pathway analyses to better understand the biological significance of the identified miRNAs. GO analysis showed that the predicted target genes were enriched in: Biological processes: Transcriptional regulation by RNA polymerase II, signal transduction, lipid metabolism, etc; Cellular components: Cell membrane, cell nucleus, cytoplasm, exosome, etc; Molecular functions: Protein binding, RNA binding, RNA polymerase II-specific DNA-binding transcription factor activity, etc. (**Figure 3B**). KEGG pathway analysis revealed that the predicted target genes were significantly enriched in pathways related to T2DM, including: Autophagy, cAMP signaling pathway and ErbB signaling pathway (**Figure 3C**). The top 5 most significant pathways identified were: Axon guidance; Autophagy; Proteoglycans in cancer; cAMP signaling pathway; ErbB signaling pathway (**Figure 3D**).

Additionally, we constructed a Protein-Protein Interaction (PPI) network for the target genes of hsa-let-7g-5p, PC-3p-13356, and hsa-miR-6505-5p using the STRING database (**Figure 3E**). The hub gene analysis of the predicted target genes was conducted following the method described by Szydełko et al. (17), and revealed the following top 10 hub genes: PTEN; AGO2; SKP1; SMAD2; EZH2; TRRAP; PIK3CA; SUMO2; GCC1; MMUT. Among these, PTEN had the highest score (**Figure 3F**).

## 4 Discussion

This study compared the exosomal miRNA expression profiles in the plasma of three groups: NAO/NG (non-abdominal obesity with normal glucose), AO/NG (abdominal obesity with normal glucose), and AO/T2DM (abdominal obesity with newly diagnosed type 2 diabetes). The results revealed specific differential expression patterns of exosomal miRNAs in AO/T2DM patients. Notably, certain miRNAs exhibited a gradual increase or decrease in expression from NAO/NG to AO/NG, and finally to AO/T2DM, suggesting a potential role in the progression of abdominal obesity to T2DM.

Large amounts of evidence in recent years have suggested a crucial role of exosomal miRNAs in various metabolic processes such as glucose and lipid metabolism, insulin signaling, inflammation, and adipogenesis, and are closely related to obesity and T2DM (18). While several key studies on exosomal miRNAs have focused on systemic obesity and its link to T2DM, the specific role of exosomal miRNAs in the progression from abdominal obesity to T2DM remains unexplored. To further explore this and identify biomarkers, five plasma exosomal miRNAs were screened for extended sample validation. The results showed that the expression level of exosomal hsa-let-7g-5p exhibited a stepwise increase across the three groups, consistent with the screening results. Further clinical correlation analysis revealed that exosomal hsa-let-7g-5p was positively correlated with VFA, FBG, HOMA-IR, and LDL-C, while it was negatively correlated with HDL-C and HOMA-β, indicating that exosomal hsa-let-7g-5p is closely associated with visceral fat accumulation and glucose-lipid metabolism. Similar to our results, Lirun et al. (19) found that serum let-7 levels significantly decreased after Roux-en-Y gastric bypass surgery in obese T2DM patients. In line with this, Zhu et al. (20) observed an increase in let-7 expression during the differentiation of 3T3-L1 preadipocytes, demonstrating that let-7 regulates adipogenesis by specifically targeting the transcription factor HMGA2. Furthermore, Jiang et al. (21) reported that the expression of let-7 in the skeletal muscles of T2DM patients was significantly higher than that in individuals with normal glucose tolerance, and it was closely related to IR. Animal studies further support the findings that overexpression of let-7 in mouse skeletal muscles is associated with IR and impaired glucose tolerance (22). In contrast to our study, Dogan et al. (23) found no significant changes in the expression of exosomal let-7g-5p derived from visceral fat tissue in obese and T2DM patients compared to non-obese individuals. The reason for the differences in results between our study and previous studies may be due to ethnic heterogeneity (this study was conducted in a Chinese population, while previous studies were conducted in Turkish populations), differences in obesity diagnostic criteria (our study used VFA to diagnose abdominal obesity, while previous studies used BMI), and differences in sample types (our study used plasma exosomes, while previous studies primarily used fat tissue exosomes), among other factors. Future research should consider these factors to clarify the role of extracellular vesicle miRNAs in metabolic diseases. Furthermore, it is noteworthy that a novel exosomal miRNA, PC - 3p - 13356, was identified. It exhibited a stepwise increase in expression among the study population. Clinical correlation analysis further revealed that exosomal PC - 3p - 13356 was significantly associated with visceral fat area and glucose-lipid metabolism indicators. The reason for the observed increase in PC-3p-13356 content in serum EVs from patients with obesity and T2DM is unclear. Given that the expression levels of exosomal miRNAs can serve as potential biomarkers reflecting the metabolic state of the body, we further evaluated the diagnostic value of exosomal hsa-let-7g-5p and PC-3p-13356 in abdominal obesity-related T2DM. ROC curve analysis showed that exosomal hsa-let-7g-5p and PC-3p-13356 displayed good biomarker potential in distinguishing between AO/NG and AO/T2DM groups. Previous studies have confirmed that let-7 can serve as a potential biomarker for chronic kidney disease in hypertension patients (24), prognosis in pancreatic cancer patients (25), and for children with type 1 diabetes (26). These findings further support the potential of let-7 as a disease biomarker. In conclusion, the specific dynamic changes in plasma exosomal hsa-let-7g-5p and PC-3p-13356 may play important roles in the development and progression of the disease from NAO/NG to AO/NG and AO/T2DM, and could serve as potential biomarkers for monitoring the risk of T2DM in abdominal obesity populations.

In addition, plasma exosome hsa-miR-6505-5p was found as a microRNA that is potentially specifically expressed in abdominal obesity-associated type 2 diabetes. Although the key role of hsa-miR-6505-5p in obesity and T2DM remains unknown, our clinical correlation analysis of exosomal hsa-miR-6505-5p showed that it was significantly positively correlated with VFA, FBG, HbA1c, and HOMA-IR, while negatively correlated with HOMA-β. Further ROC curve analysis showed that, among the independent predictive indicators of the candidate biomarkers in this study, exosomal hsa-miR-6505-5p had the highest AUC score as an independent predictor (AUC: 0.878). Moreover, when combined with hsa-let-7g-5p and PC-3p-13356 in a joint diagnostic model, the AUC of the three-marker combined model reached 0.891, indicating that exosomal hsa-miR-6505-5p has good potential to distinguish between AO/NG and AO/T2DM groups, and suggesting that it could serve as a valuable biomarker for early identification of T2DM high-risk populations.

To further elucidate the molecular mechanisms through which exosomal miRNAs drive the progression of T2DM in abdominal obesity, this study performed KEGG pathway enrichment analysis. The results showed that exosomal hsa-let-7g-5p, PC-3p-13356, and hsa-miR-6505-5p were significantly enriched in metabolic pathways such as autophagy, cAMP signaling, and ErbB signaling. The study found that the cAMP signaling pathway can promote insulin release through mechanisms including membrane depolarization, intracellular calcium concentration ([Ca2+]c) signal transduction, recruitment of insulin granules to the plasma membrane, and activation of exocytosis (27). The cAMP signaling pathway also plays an important role in fat metabolism. Activation of the β-adrenergic receptor/cAMP signaling pathway in adipocytes can promote triglyceride breakdown, a process negatively regulated by phosphodiesterase (28). Recent research has shown that autophagy is closely associated with the onset and progression of T2DM (29). Studies on Atg7-deficient mice, which lack autophagy, showed that autophagy dysfunction leads to a reduction in pancreatic β-cell numbers, impaired glucose tolerance, and decreased insulin secretion capacity (30). The ErbB signaling pathway, as an important node in regulating metabolic homeostasis, participates in the regulation of glucose and lipid metabolism through processes such as β-cell proliferation and regeneration, lipogenesis, and insulin resistance (31–33). To further explore the association between the predicted target genes and identify key genes, this study constructed a PPI network and found that phosphatase and tensin homolog (PTEN) was the highest-scoring gene in the hub gene analysis. PTEN, as a key negative regulator of insulin signaling, is closely related to insulin signal transduction abnormalities and disturbances in glucose homeostasis regulation (34, 35). Recent studies have shown that PTEN expression is significantly reduced in the adipose tissue of obese patients with diabetes compared to obese individuals without diabetes (36). Animal experiments have shown that PTEN overexpression in mice results in increased insulin sensitivity, lower blood glucose and insulin levels, and significant resistance to diet-induced fatty liver; additionally, these mice displayed characteristics of improved metabolism, such as increased energy expenditure, reduced fat mass, and enhanced brown adipose tissue activity (37). *In vitro* studies have also found that silencing miR-217 can significantly increase podocyte glucose uptake and improve insulin resistance by activating the PTEN-mediated autophagy pathway (38). In conclusion, we speculate that these three miRNAs may play a role in the development of abdominal obesity-related T2DM by targeting key genes such as PTEN and mediating autophagy and other signaling pathways.

In conclusion, our study is the first to reveal a specific dynamic expression profile of plasma exosomal miRNAs in non-abdominal obesity with normal glucose metabolism, abdominal obesity with normal glucose metabolism, and abdominal obesity-related T2DM patients within the Chinese population. We found that plasma exosomal hsa-let-7g-5p, PC-3p-13356, and hsa-miR-6505-5p are associated with the progression of the disease and may serve as potential biomarkers for monitoring the risk of T2DM in individuals with abdominal obesity. However, there are several limitations to this study. First, it is a cross-sectional study; second, the sample size is limited; third, the exact source of plasma exosomes was not identified; fourth, although we performed functional and pathway enrichment analyses to explore the potential biological significance of differentially expressed miRNAs, further validation in both *in vitro* and *in vivo* experimental models is needed to confirm the role of miRNA target genes in the pathogenesis of abdominal obesity-related T2DM. In summary, our study provides new directions for the early identification of high-risk groups for T2DM, contributing to early diagnosis and treatment of T2DM, and offering novel insights for precision treatment and early disease reversal in abdominal obesity-related T2DM.

## 5 Conclusion

This study suggests that circulating exosomal miRNAs may serve as potential biomarkers for monitoring the risk of T2DM development in individuals with abdominal obesity. These miRNAs could regulate key hub genes like PTEN and mediate metabolic pathways such as autophagy, cAMP signaling, and ErbB signaling, thereby contributing to the pathogenesis of abdominal obesity-related T2DM. However, the precise mechanisms by which these miRNAs influence disease progression need further validation through additional studies.

## Data Availability

The datasets presented in this study can be found in online repositories. The names of the repository/repositories and accession number(s) can be found in the article/**Supplementary Material**.
