# Supplementary material for: Plasma-derived exosomal miRNAs as potentially novel biomarkers for type 2 diabetes mellitus with abdominal obesity
Source: Front Endocrinol (Lausanne). 2025 Nov 28;16:1656132. doi: 10.3389/fendo.2025.1656132 (PMC12698432; doi:10.3389/fendo.2025.1656132)
Supplement: Supplementary file 1 [file DataSheet1.docx]

Supplementary Material

# Supplementary Table

Supplementary Table1 Primer sequences for RT-qPCR

| **miRNA** | **Forward sense** |
| --- | --- |
| hsa-let-7g-5p | TGAGGTAGTAGTTTGTACAGTT |
| PC-3p-13356 | CAGAAACCAGAATTACTTTTGC |
| hsa-miR-6505-5p | TTGGAATAGGGGATATCTCAGC |
| hsa-miR-1229-3p | CTCTCACCACTGCCCTCCCACAG |
| hsa-miR-4750-5p | CTCGGGCGGAGGTGGTTGAGTG |

Supplementary Table 2 Exosomal miRNAs only in the AO/NG group

| **miRNA_ID** | **log2 Fold change** | **Regulation** | ***p*** |
| --- | --- | --- | --- |
| hsa-miR-147b-3p | 2.86 | up | 0.01 |
| hsa-miR-10401-3p | 2.43 | up | 0.00 |
| hsa-miR-4488 | 2.17 | up | 0.04 |
| hsa-miR-659-5p | 1.92 | up | 0.01 |
| hsa-miR-885-5p | 1.65 | up | 0.03 |
| hsa-miR-619-p5 | 1.61 | up | 0.02 |
| hsa-miR-378d | 1.48 | up | 0.00 |
| hsa-miR-582-3p | 1.38 | up | 0.02 |
| hsa-miR-642a-3p | 1.26 | up | 0.02 |
| hsa-miR-375-3p | 1.17 | up | 0.01 |
| hsa-miR-150-3p | 1.13 | up | 0.02 |
| PC-3p-14618 | 1.11 | up | 0.01 |
| hsa-let-7e-3p | 1.08 | up | 0.03 |
| hsa-miR-1306-5p | -1.18 | down | 0.02 |
| hsa-miR-301b-3p | -1.28 | down | 0.01 |
| hsa-miR-6837-3p | -2.31 | down | 0.04 |

Supplementary Table 3 Exosomal miRNAs only in the AO/T2DM group

| **miRNA_ID** | **log2 Fold change** | **Regulation** | ***p*** |
| --- | --- | --- | --- |
| hsa-miR-6505-5p | 2.47 | up | 0.01 |
| PC-3p-13356 | 1.32 | up | 0.01 |
| hsa-miR-128-1-5p | 1.12 | up | 0.04 |
| hsa-miR-5100-p3 | -1.36 | down | 0.00 |
| hsa-miR-6721-5p | -1.46 | down | 0.04 |
| hsa-miR-933 | -2.03 | down | 0.04 |
| hsa-miR-4750-5p | -2.29 | down | 0.04 |

Supplementary Table 4 Specific exosomal miRNAs that continuously changed

in three groups

| **miRNA_ID** | **Regulation** | ***p*** |
| --- | --- | --- |
| hsa-miR-1229-3p | up | 0.00 |
| hsa-let-7g-5p | up | 0.02 |
| PC-3p-13356 | up | 0.02 |
| hsa-miR-6505-5p | up | 0.02 |
| hsa-miR-4750-5p | down | 0.04 |

Supplementary Table 5 Validation results of differentially expressed miRNAs

in plasma exosomes

| **miRNA** | **NAO/NG** | **AO/NG** | **AO/T2DM** | ***p*** |
| --- | --- | --- | --- | --- |
| hsa-let-7g-5p | 0.78(0.60,0.97) | 1.28(1.09,2.18)^a^ | 3.11(2.06,6.15)^ab^ | <0.001 |
| PC-3p-13356 | 0.85(0.62,1.39) | 2.17(0.81,11.88)^a^ | 9.51(6.21,20.73)^ab^ | <0.001 |
| hsa-miR-6505-5p | 0.83(0.57,1.00) | 0.94(0.69,1.36) | 2.56(2.06,4.42)^ab^ | <0.001 |
| hsa-miR-1229-3p | 1.17(0.86,2.25) | 0.30(0.24,0.80)^a^ | 0.79(0.58,1.15)^b^ | <0.001 |
| hsa-miR-4750-5p | 0.96(0.56,4.13) | 0.68(0.23,0.93) | 1.27(0.67,1.81) | 0.068 |

Notes: **NAO/NG**: Non-abdominal obesity with normal glucose metabolism group; **AO/NG**: Abdominal obesity with normal glucose metabolism group; AO/T2DM: Abdominal obesity with type 2 diabetes group. ^a^*p <* 0.05 (vs NAO/NG); ^b^*p <* 0.05 (vs AO/NG).

# Supplementary Figures

**Supplementary Figure 1.** AO/NG specific differential exosomal miRNAs screening flowchart

**
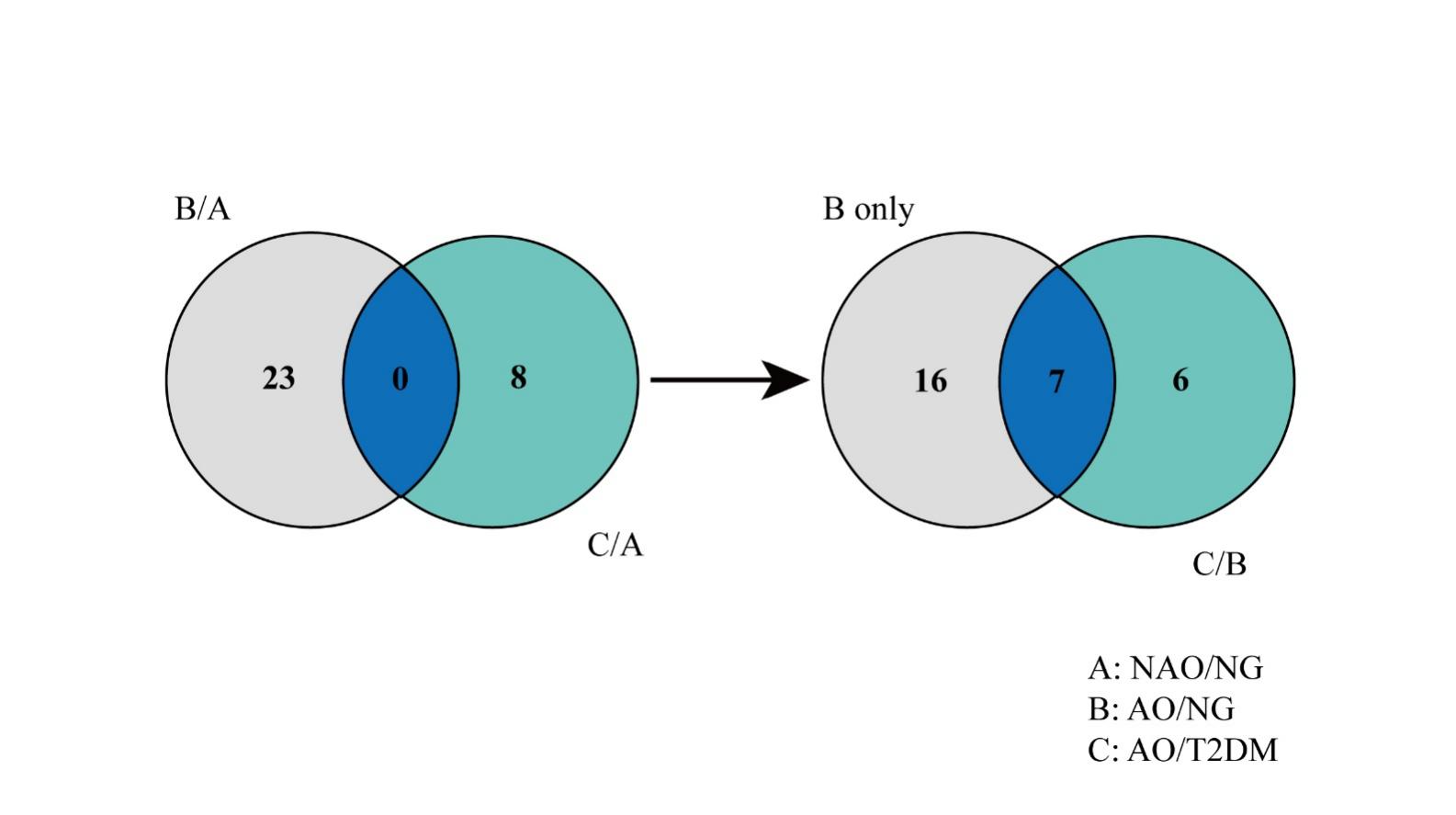
**

**Supplementary Figure 2.** AO/T2DM specific differential exosomal miRNAs screening flowchart


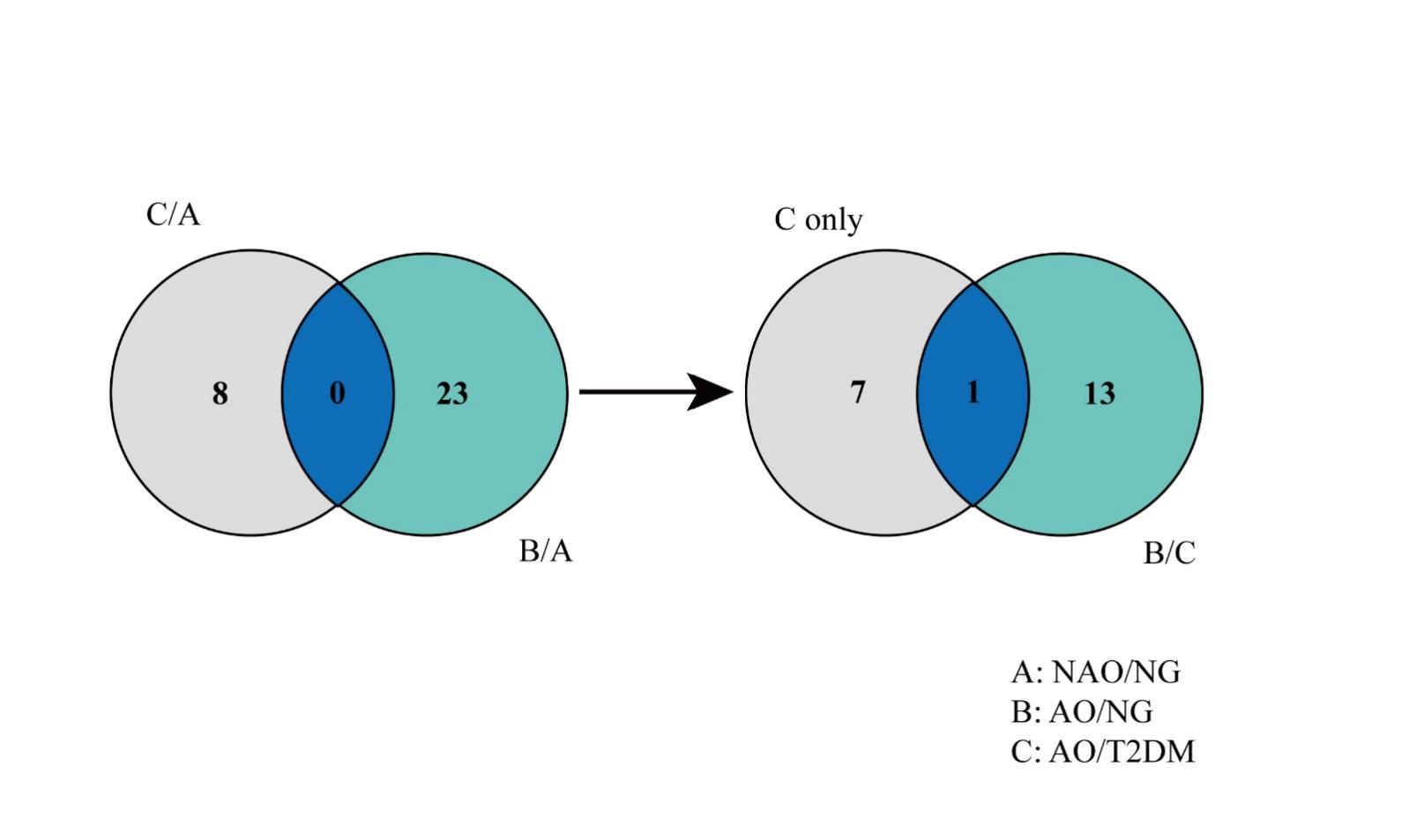


**Supplementary Figure 3.** Cluster analysis of differentially expressed exosomal miRNAs in three groups sequentially.

The horizontal axis of the clustering analysis heatmap represents the samples, and the vertical axis represents the miRNAs. Different colors indicate different miRNA expression levels, with the color gradient from blue to white to red representing expression levels from low to high. Red indicates high expression of miRNAs, while dark blue indicates low expression of miRNAs.


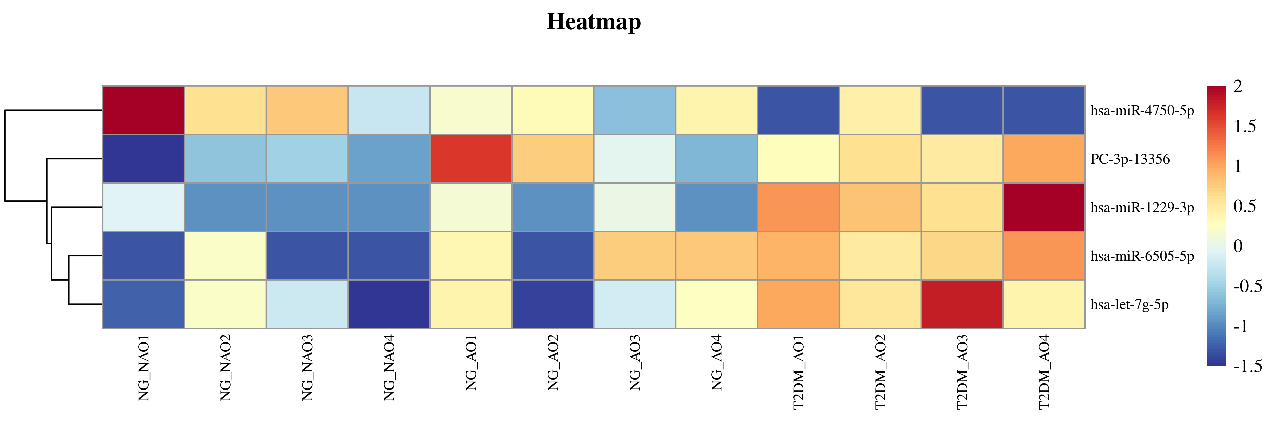


**Supplementary Figure 4.** Heatmap of the correlation between plasma exosomal
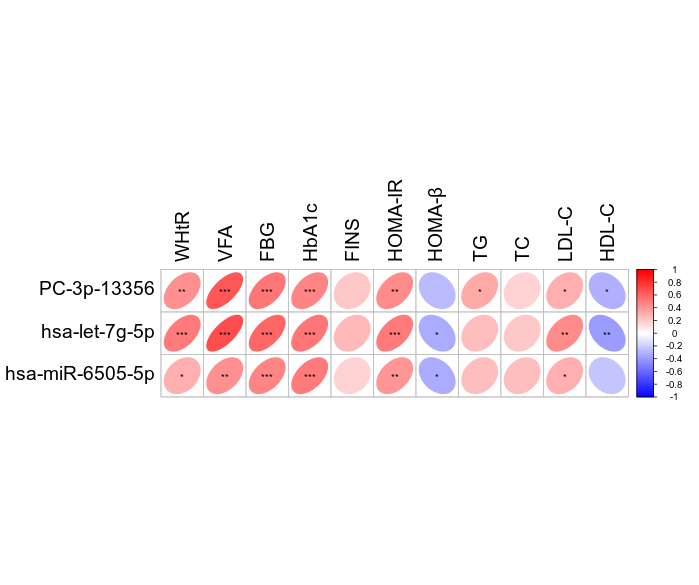
miRNA and clinical indicators. **P <* 0.05，***P <* 0.01，****P <* 0.01
